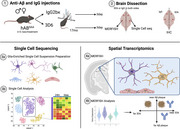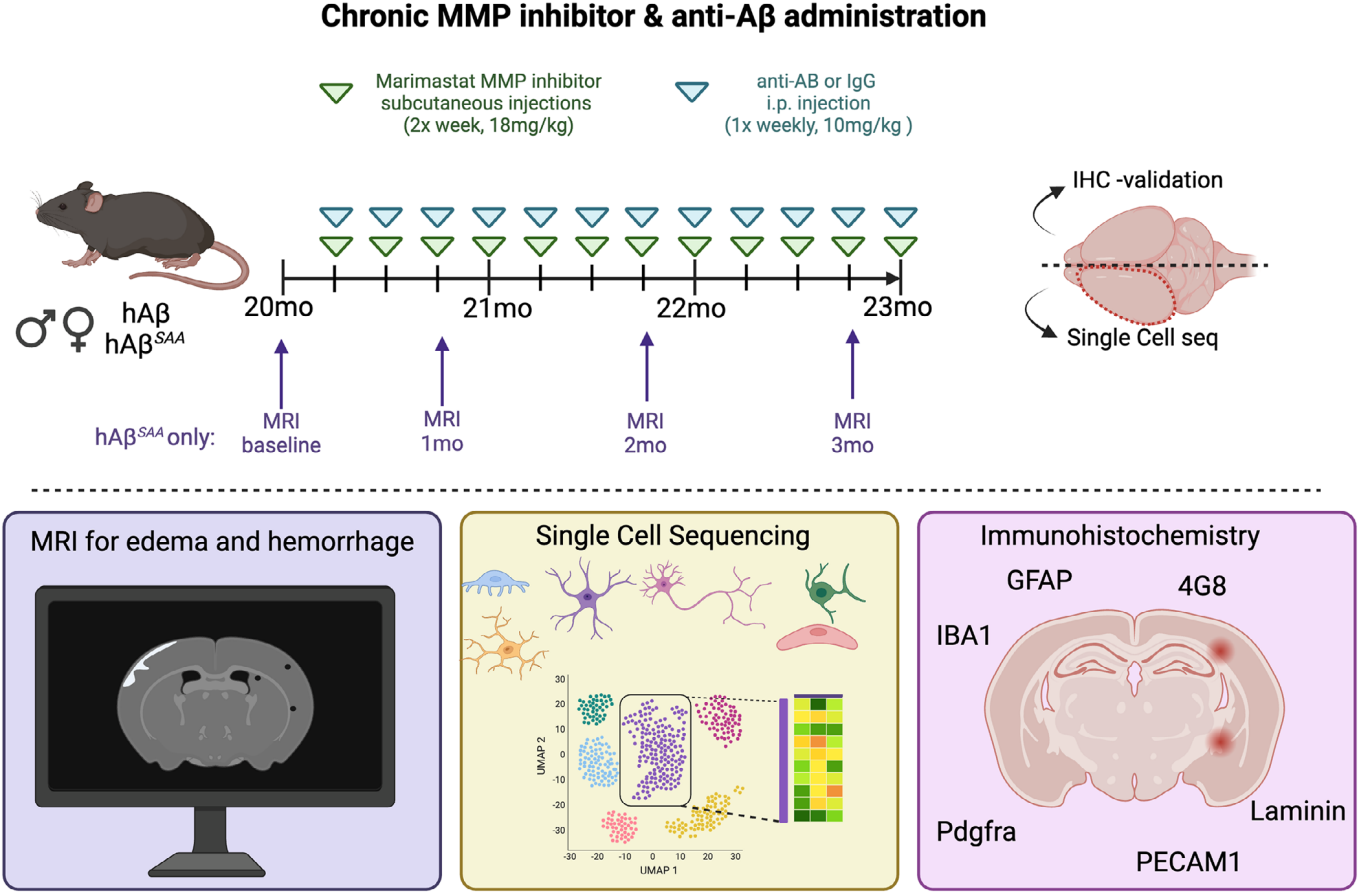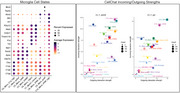# Profiling the immune response to anti‐Aβ immunotherapy

**DOI:** 10.1002/alz70855_106875

**Published:** 2025-12-24

**Authors:** Kate E. Foley, Katelynn E. Krick, Erica M. Weekman, Donna M. Wilcock

**Affiliations:** ^1^ Indiana University School of Medicine, Stark Neurosciences Research Institute, Department of Neurology, Indianapolis, IN, USA; ^2^ Indiana University School of Medicine, Stark Neurosciences Research Institute, Department of Anatomy, Cell Biology, and Physiology, Indianapolis, IN, USA

## Abstract

**Background:**

As patients receive approved anti‐Aβ immunotherapies the critical need to understand the effects of anti‐Aβ antibody exposure on the brain is of extreme priority. We sought to identify immune changes in response to acute and chronic anti‐Aβ antibody to determine potential causes of neuroinflammation that may lead to ARIA (amyloid‐related imaging abnormalities). Further, we investigated whether modifying the activity of MMP9, a key matrix metalloprotease that increases with anti‐Aβ antibody treatment, can reduce anti‐Aβ cerebrovascular disruption.

**Method:**

To understand cellular responses due to acute anti‐Aβ antibody exposure, we intracranially injected 17mo humanized Aβ (hAβ) mice with and without familial amyloid mutations (Swedish, Arctic, Austrian; hAβ^SAA^) with anti‐AB 3D6 or control IgG for 3 days (Figure 1). This acute study evaluates early transcriptional changes that occur in glial and peripheral immune cells, using single cell sequencing (SCseq), coupled with spatial transcriptomics to identify acute molecular responses. To evaluate chronic anti‐Aβ exposure, we injected 3D6 intraperitoneally for 3 months starting at 20mo (Figure 2). We evaluated ARIA through longitudinal MRI, cellular responses via SCseq, spatial transcriptomics, and IHC. To investigate the effect of MMP9 inhibition and neuroinflammation on ARIA risk, we co‐injected these mice with Marimastat, a small molecular inhibitor of several MMPs throughout this study.

**Result:**

The acute SCseq study revealed diverse microglial cell states in response to injection (Figure 3, left). There were sex and treatment specific responses of various microglia sub‐populations. There was a sex‐specific effect on the number of differentially expressed genes across microglia subtypes with an emphasis on downregulation of cellular programs, particularly in translation and neuronal support suggesting early responses to anti‐Aβ antibody affects immune cell prioritizations (data not shown). We found stark differences between anti‐Aβ and IgG responses in ligand‐receptor communications involving microglia, suggesting an altered immune response (Figure 3, right). The chronic study analysis is ongoing.

**Conclusion:**

These data lay the groundwork for understanding the brain's unique response to anti‐Aβ antibody and early neuroinflammation.